# The Oriental Fruit Fly, *Bactrocera dorsalis*, in China: Origin and Gradual Inland Range Expansion Associated with Population Growth

**DOI:** 10.1371/journal.pone.0025238

**Published:** 2011-10-03

**Authors:** Xuanwu Wan, Francesco Nardi, Bin Zhang, Yinghong Liu

**Affiliations:** 1 Chongqing Key Laboratory of Entomology and Pest Control Engineering, College of Plant Protection, Southwest University, Chongqing, China; 2 Department of Evolution Biology, University of Siena, Siena, Italy; Aarhus University, Denmark

## Abstract

The oriental fruit fly, *Bactrocera dorsalis*, expanded throughout mainland China in the last century to become one of the most serious pests in the area, yet information on this process are fragmentary. Three mitochondrial genes (*nad1*, *cytb* and *nad5*) were used to infer the genetic diversity, population structure and demographic history of the oriental fruit fly from its entire distribution range in China. High levels of genetic diversity, as well as a significant correspondence between genetic and geographic distances, suggest that the invasion process might have been gradual, with no associated genetic bottlenecks. Three population groups could be identified, nevertheless the overall genetic structure was weak. The effective number of migrants between populations, estimated using the coalescent method, suggested asymmetric gene flow from the costal region of Guangdong to most inland regions. The demographic analysis indicates the oriental fruit fly underwent a recent population expansion in the Central China. We suggest the species originated in the costal region facing the South China Sea and gradually expanded to colonize mainland China, expanding here to high population numbers.

## Introduction

The oriental fruit fly, *Bactrocera dorsalis* (Hendel), is one of the most important pests on fruits and vegetables across South East Asia and the Pacific region [1]. Being highly polyphagous, the oriental fruit fly can infest a wide variety of fruit crops, such as citrus, mandarin, peach and mango [2], [3], and induce significant economic losses through direct fruit damage, fruit drop and export limitations associated to quarantine restrictions. Furthermore, due to its broad host range, relatively ample climate tolerance, high reproductive potential and dispersal capacity [4], the oriental fruit fly is considered to have a high invasive potential.

The oriental fruit fly has significantly expanded its geographic distribution in the last century. Early records report its presence in 1912 in Taiwan [5]. Henceforth, this species colonized different areas of the Asian and Pacific regions, such as India, Pakistan, Nepal, Vietnam, Laos, Burma, Thailand, Sri Lanka, the Northern Mariana Islands (eradicated), Hawaii, Guam, with transient appearances in California and Florida [1], [2]. The potential distribution analysis showed that the oriental fruit fly is likely to expand in the next future North and South-ward into areas currently cooler [2].

After its initial recognition in Taiwan, the oriental fruit fly was reported in 1934 on Hainan Island, China [6], and sparsely in disjointed areas of Southern China until the 1970s. Since the 1980s the population size of the oriental fruit fly increased quickly and the distribution area expanded rapidly to cover most areas south of the 26N parallel. In the last decade the oriental fruit fly expanded across the Yangtze River to reach the 32N parallel [7]–[9] and is expected to expand further North [10].

Despite the economic and ecological threats associated with the invasion of the oriental fruit fly, data on its phylogeography are scarce. An early study investigated the relationship between two laboratory and three wild populations [11]. Afterwards, studies on *Bactrocera dorsalis* population genetics and phylogeography mostly addressed specific and/or geographically limited issues [12]–[17], until a first integrated attempt to study this species in a substantial part of its range conducted by Aketarawong et al. [18]. Aketarawong and colleagues suggested China as the origin of fly populations in South-East Asia and could describe a well supported pattern of expansion from the region of Guangdong to this latter area. Nevertheless, their study included only two Chinese populations, from Guangdong and Taiwan, limiting their possibility to study the dispersal of the oriental fruit fly in mainland China.

Mitochondrial DNA, due to its accelerated rate of evolution, short coalescence time and simple maternal inheritance, has been used as a marker of choice for historical phylogeography and complements well with the information provided by microsatellite markers. Thanks to the possibility to reconstruct evolutionary relationships among haplotypes, the mitochondrial DNA is particularly informative to reconstruct historical processes, such as the identification of the region of origin of a species, the pathways of invasion and historical demography, and has been repeatedly used to study the spread of alien species [19]–[26]. Furthermore, mitochondrial markers have repeatedly been used in fruit flies, such as the olive fly [27]–[29], the pumpkin fruit fly [30], the melon fruit fly [31] and the Mediterranean fruit fly [32]–[34]. Limitation to the use of mitochondrial markers is that the entire molecule is non recombining and inherited as a single locus, henceforth limiting the possibility to average across markers to take into account the random nature of the coalescence process.

With this study we specifically focus on Chinese populations, previously identified as a likely source for the species, to tackle the issue of the origin and range expansion of the oriental fruit fly. Specifically at issue are: a) a first exploration of Chinese diversity and definition of local genetic groups; b) the reconstruction of the major routes of expansion in mainland China and the definition of the demographic profile associated with the expansion, and c) the identification of the region of origin of the species and a re-evaluation of the different hypotheses proposed in the literature.

## Materials and Methods

### Sampling, DNA extraction and sequencing

**Figure 1:**
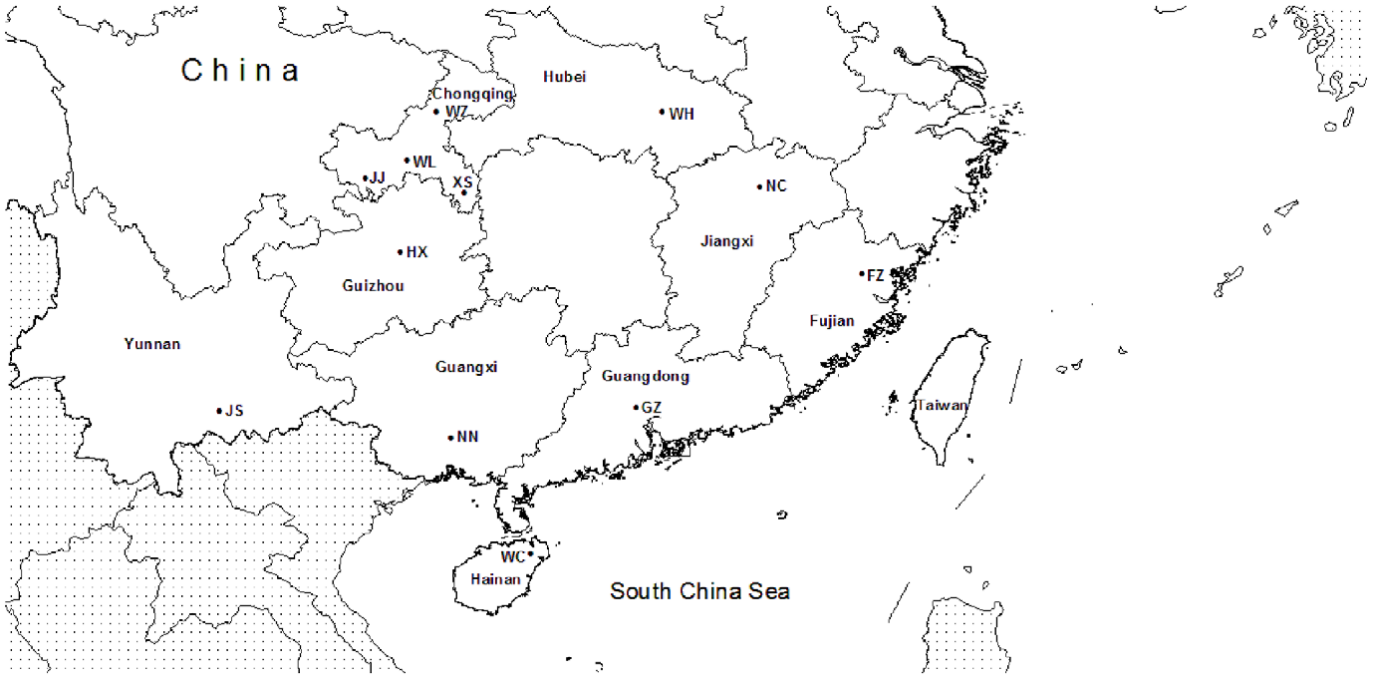
Collecting sites. See Tab. 1 for complete collection information.

Samples of *Bactrocera dorsalis* were collected from 12 locations covering the entire distribution range in China during years 2009–2010 using traps baited with Methyl Eugenal (Tab. 1; Fig. 1). Specimens were preserved in 95% ethanol at −20°C until processing.

**Table 1:** Sampling locations.

Location, province	Code	n°	Longitude (E)	Latitude (N)	year
Fuzhou, Fujian	FZ	17	119°28′	26°15′	2009
Wenchang, Hainan	WC	16	110°76′	19°68′	2010
Nanning, Guangxi	NN	20	108°46′	22°78′	2010
Huaxi, Guizhou	HX	9	106°67′	26°44′	2009
Wuhan, Hubei	WH	20	114°36′	30°48′	2009
Guangzhou, Guangdong	GZ	19	113°28′	23°18′	2009
Nanchang, Jiangxi	NC	20	115°79′	28°62′	2009
Jianshui, Yunnan	JS	20	102°82′	23°70′	2010
Jiangjin, Chongqing	JJ	20	106°25′	29°08′	2009
Wanzhou, Chongqing	WZ	20	108°50′	30°75′	2009
Wulong, Chongqing	WL	20	108°97′	28°42′	2009
Xiushan, Chongqing	XS	20	107°02′	29°30′	2009

n°, number of individuals studied.

Total DNA was extracted using the DNeasy Blood and Tissue Kit (QIAGEN) from 221 individual specimens. Three fragments of the mitochondrial genome (574 bp of *nad1*, 750 bp of *cytb* and 658 bp of *nad5*) were amplified from each and all individuals using primer pairs nad1-F (5′-TTTAGTTGCTTGGTTGTGTATTCC-3′)/nad1-R (5′-GAAAAAGGTAAAAAACTCTTTCAAGC-3′), cytb-F (5′-AACTCTTCACGCCAACGG)/cytb-R (5′-GGTCGTGCTCCAATTCAT-3′) and nad5-F (5′-TAACCCAATACACCTCCT-3′), nad5-R (5′-GGTAACTGCTGGGGTTTA-3′). Primers for *nad1* are from Nardi et al. (2005), primers for *cytb* and *nad5* were designed based on available complete mitochondrial genome sequences of tephritids, including *B.dorsalis* [35]. Amplifications were carried out for 35 cycles of 30″ at 94°C, 1′ at 54°C, 1′ 30″ at 72°C, with an initial denaturation step of 5′ at 94°C and a final extension of 10′ at 72°C. Amplification products were purified and sequenced by Invitrogen Biotechnology Co. (Shanghai, China) on both strands using PCR primers. After manual correction and assembly, unique sequences were deposited in GenBank under accession numbers JF521024-JF511166 (*cytb*), JF521167-JF521298 (*nad1*) and JF521299-JF521440 (*nad5*).

### Data analysis

Sequences were aligned using ClustalX (ver. 2.0) [36] and unique haplotypes were identified in ARLEQUIN (ver. 3.5) [37]. Descriptive statistics (number of variable sites, number of haplotypes, haloptype diversity, nucleotide diversity, average number of nucleotide difference between haplotypes) were calculated in Dnasp (ver. 5.0) [38].

Spatial analysis of molecular variance was performed using SAMOVA (ver. 1.0) [39] to identify population groups, with concatenated sequences of the three genes for each individual, longitude and latitude information as input data. The most supported number of groups (K) was determined by repeating the analysis with K ranging from 2 to 6 and selecting the subdivision scheme associated with highest F_CT_. An AMOVA hierarchical analysis of variance was performed using ALEQUIN to partition total variance in its components among groups, among populations and within populations, based on the groups inferred by the SAMOVA analysis. The correlation between genetic (F_ST_/1-F_ST_) and geographic distance matrices (in ln scale) [40] was tested using the IBDWS web service [41] with 10000 randomizations. Median-joining networks of haplotypes of each of the three genes were constructed using NETWORK (ver. 4.6) [42], [43] to study the evolutionary relationships among haplotypes.

The extent of gene flow between population pairs was studied using the coalescent-based strategy implemented in MIGRATE (ver. 3.2.7) [44]. To determine if there was asymmetrical gene flow between populations, the mutation scaled effective immigration rate (M = m/μ) entering and leaving each population per generation and the mutation scaled effective population size (Θ = N_e_μ) were jointly estimated applying the Bayesian search strategy. N_e_m was calculated by multiplying these latter values. Four independent runs of MIGRATE, each consisting of one long chain of 100,000,000 generations with the initial 10,000 excluded as burn-in of the analysis, were conducted to assess consistency in the results, changing seed number at each run.

The demographic history of all populations pooled together and of each of the three population groups identified by the SAMOVA analysis was studied using mismatch distributions in ARLEQUIN. Tajimas'*D* ad Fu's *F_S_* were calculated to test for neutrality. Population size before expansion (θ_0_), population size after expansion (θ_1_), population expansion time (τ), and sum of squared deviation (SSD) between observed and expected mismatch distributions were similarly calculated. All parameters were tested against the expected values under the hypothesis of a recent population expansion based on 1000 bootstrap replicates.

## Results

### Genetic diversity

Collapsing of individual sequences led to the identification of 132, 143 and 142 unique haplotypes for genes *nad1*, *cytb* and *nad5*, respectively, or 164 after concatenation of the three markers for each individual.

Basic descriptive statistics, calculated for each population based on concatenated sequences as well as each of the three genes independently, are shown in Tab.S1. The number of haplotypes per population (n) ranged from 9 to 20, 6 to 19, 8 to 20, 9 to 20 in concatenated sequences, *nad1*, *cytb* and *nad5*, respectively. Haplotype diversity (H) ranged from 0.9006 to 1, 0.8889 to 1, 0.8947 to 1 and 0.8947 to 1, nucleotide diversity (π) from 0.0093 to 0.0125, 0.0077 to 0.0147, 0.0105 to 0.0194 and 0.0081 to 0.0117, similarly in concatenated sequences, *nad1*, *cytb* and *nad5*. These figures suggest that all populations retain fairly high levels of genetic diversity.

### Genetic structure

Monitoring of F_CT_ values in the SAMOVA analysis suggested 3 as the optimal number of population groups (F_CT3_ = 0.04231). The 12 populations were clustered as follows: Jiangjin, Wulong, Wanzhou, Xiushan, Huaxi, Nanning, Wuhan, Nanchang and Jianshui; Fuzhou and Guangzhou; Wenchang. These three groups correspond to three geographically well defined regions that we refer to as Central, South-East and Southern China in the following presentation (see Fig. 1).

The AMOVA analysis revealed that a substantial portion of genetic differentiation is partitioned among groups (4.22% based on concatenated sequences, from 2.29% to 6.96% based on individual genes) and inside populations (94.95%; 92.63% to 96.89%), while genetic differentiation between populations inside each of the three groups identified is limited (0.84%; 0.4% to 1.29%) (Tab. 2). Accordingly, differentiation among groups (F_CT_) and within populations (F_ST_) are highly significant, while differentiation among populations between groups (F_SC_) was not or marginally (*nad5* only) significant (Tab. 2).

**Table 2:** Partitioning of genetic variation at different hierarchical levels.

Gene analyzed	Source of variation	d.f.	Sum of squares	Variance components	Percentage of variation	Fixation indices
concatenated sequences	Among groups	2	65.720	0.48104Va	4.22	F_CT_ = 0.04217**
	Among populations within groups	9	113.464	0.09545Vb	0.84	F_SC_ = 0.00874
	Within populations	209	2263.522	10.83025Vc	94.95	F_ST_ = 0.05054**
*nad1*	Among groups	2	25.133	0.22173Va	6.96	F_CT_ = 0.06964**
	Among populations within groups	9	28.702	0.01288Vb	0.4	F_SC_ = 0.00435
	Within populations	209	616.391	2.94924Vc	92.63	F_ST_ = 0.07369**
*Cytb*	Among groups	2	20.515	0.11292Va	2.29	F_CT_ = 0.02292**
	Among populations within groups	9	49.754	0.04054Vb	0.82	F_SC_ = 0.00842
	Within populations	209	997.677	4.77357Vc	96.89	F_ST_ = 0.03115*
*nad5*	Among groups	2	20.110	0.14676Va	4.46	F_CT_ = 0.04458**
	Among populations within groups	9	35.047	0.04251Vb	1.29	F_SC_ = 0.01351*
	Within populations	209	648.504	3.10289Vc	94.25	F_ST_ = 0.05749**

*P<0.05,

**P<0.01.

The Mantel test for correlation between genetic and geographic distances revealed a significant correlation based on the concatenated dataset (r = 0.3667; P = 0.008) (Fig.S1 A) as well as based on each individual gene (r = 0.3852, P = 0.005; r = 0.3010, P = 0.025; r = 0.3852, P = 0.008 for *nad1*, *cytb* and *nad5*, respectively) (Fig.S1 B, C, D).

### MJ networks of haplotypes

**Figure 2:**
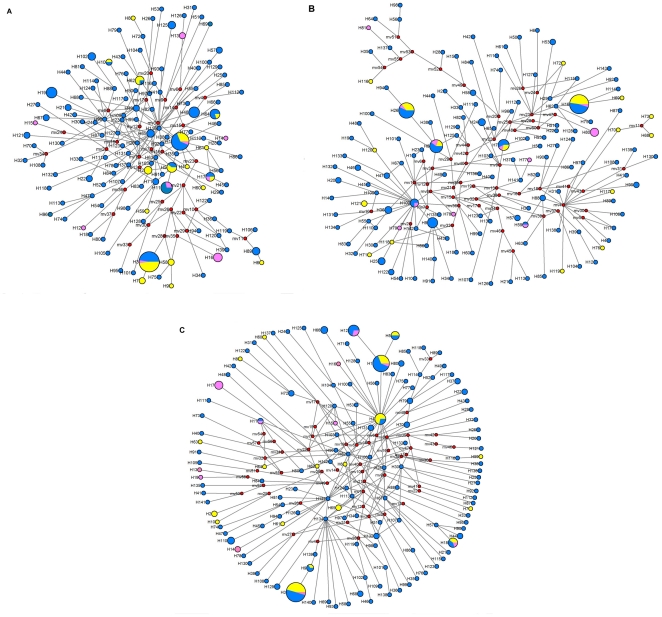
Median joining networks of haplotyps. Pie area proportional to haplotype frequency. A: *nad1*; B: *cytb*; C: *nad5*; blue: Central China group; yellow: South-East China group; pink: Southern China group; red: inferred haplotypes.

Median Joining networks reconstructed from haplotypes of the three genes are shown in Fig. 2. Networks are generally star-like with limited substructure. Some haplotypes positioned in the center of the networks are found at higher frequency in two or all three population groups (such as H77 for *nad1*, H7 and H8 for *cytb*, H4 for *nad5*), with most remaining haplotypes that are found in one single population group, generally at low frequency and connecting to central haplotypes through few mutations. Some missing haplotypes (32, 53 and 50 in *nad1*, *cytb* and *nad5*, respectively) were inferred.

### Gene flow

The effective mutation scaled population size was estimated for each population and the amount of mutation scaled immigration rate in both directions was estimated for all 66 population pairs (Tab.S2). High levels of migration rate were detected among populations, ranging from 87.6 (Xiushan to Guangzhou) to 853.0 (Guangzhou to Fuzhou). Migration rate was generally symmetrical in population pairs, i.e. the immigration rate was at par with the emigration rate. Some instances of asymmetric migration rate were nevertheless observed, based on non overlapping 95% HPD intervals for M in the two directions, namely from Guangzhou to Fuzhou, Wenchang, Wuhan, Jiangjin, Wanzhou and Xiushan. Expressed in terms of N_e_m, average gene flow inside population groups is 29.442 (range 0.778–51.673) and across population groups is 17.370 (0.322–56.664), i.e. high levels of overall gene flow are observed.

### Demographic history

Significantly negative values of Tajimas'*D* (−2.1644, P = 0.0004) and Fu's *F_S_* (−23.6507, P = 0.0056) based on concatenated sequences as well as on individual genes (see Tab. 3) indicated that the whole set of *B.doraslis* samples studied here did not fit a simple model of neutral evolution. These same estimators calculated for the three population groups indicate that South and South-East China have values of *D* and *F_S_* compatible with neutrality, while Central China has values of *D* and *F_S_* significantly deviating from neutrality (Tab. 3), suggesting Central China as the responsible for the overall disequilibrium.

**Table 3:** Parameters of demographic history of the collated 12 populations and each of three population groups independently.

Gene	Group	θ_0_	Θ_1_	τ	*D*	*Fs*	SSD
Conc. sequences	All	0.007	89.575	24.453	−2.1644**	−23.6507**	0.0016
	South-East	0.004	83.203	25.363	−0.8490	−0.8565	0.0177*
	South	0.064	96.464	26.805	2.1213	−0.7214	0.0353*
	Central	0.019	99.248	22.164	−2.2152**	−23.7562**	0.00170
*nad1*	All	1.368	46.094	5.340	−1.9658**	−24.8111**	0.0013
	South-East	0.002	10.996	7.660	−0.7696	−4.1671	0.1387
	South	0.000	41.133	10.320	−0.5318	−1.0680	0.0586*
	Central	0.991	85.156	5.236	−2.0381**	−24.9918**	0.0009
*Cytb*	All	0.009	63.047	10.311	−2.1575**	−24.188**	0.0011
	South-East	0.007	40.000	11.129	−0.8292	−2.9989	0.1806
	South	0.000	66.719	9.666	−0.0260	−0.6813	0.0449*
	Central	0.012	59.021	10.271	−2.1945**	−24.2877**	0.0008
*nad5*	All	0.011	78.594	7.656	−2.1832**	−24.7418**	0.0006
	South-East	0.000	29.727	7.928	−0.7925	−3.7418	0.0387**
	South	0.002	59.336	7.424	−0.88428	−1.6088	0.0196
	Central	0.000	99999	5.744	−2.2348**	−24.9026**	0.0294

θ_0_: effective population size before expansion; θ_1_: effective population size after expansion; τ: population expansion time; *D*: Tajiama's *D*; *F_S_*: Fu's *F_S_*; SSD: sum of squared deviations between observed and expected mismatch distribution under a sudden expansion model;

*P<0.05;

**P<0.01.

**Figure 3:**
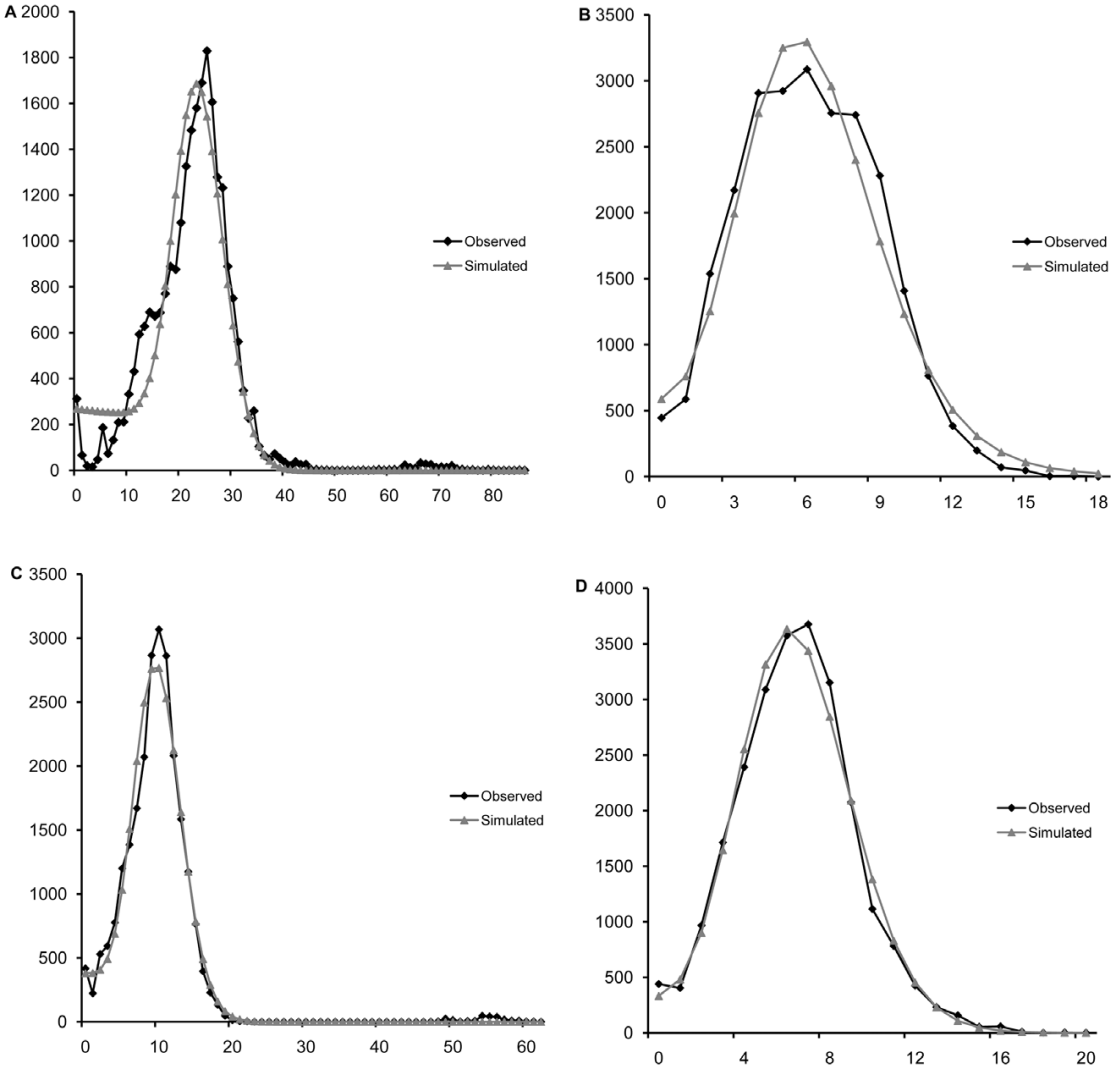
Observed and simulated mismatch distributions of entire sample. A, B, C and D are for concatenated sequences, *nad1*, *cytb* and *nad5*, respectively, the horizontal axis represents the number of pairwise differences, the vertical axis represents the relative frequency.

**Figure 4:**
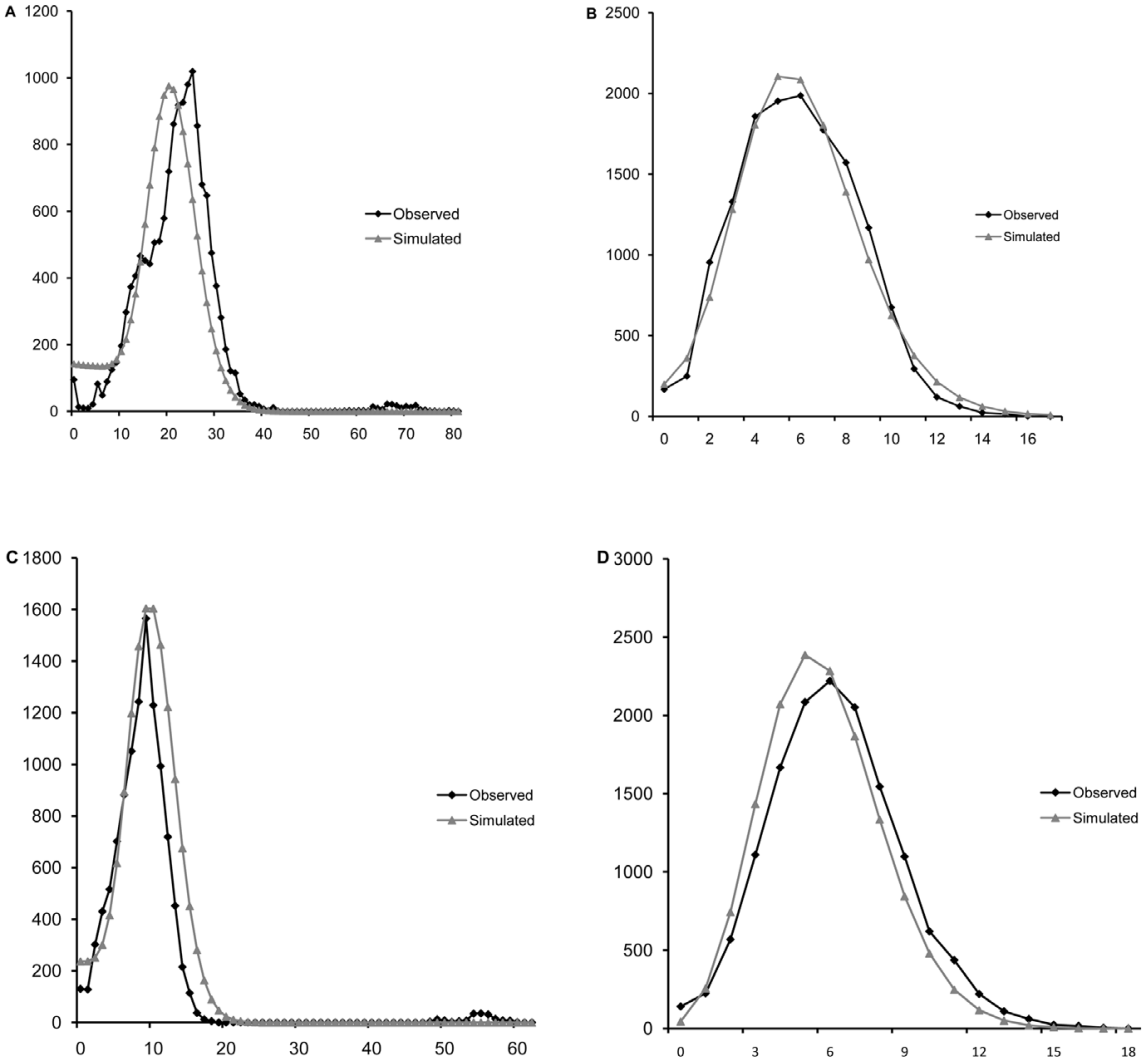
Observed and simulated mismatch distributions of Central China group only. A, B, C and D are for concatenated sequences, *nad1*, *cytb* and *nad5*, respectively, the horizontal axis represents the number of pairwise differences, the vertical axis represents the relative frequency.

The mismatch distribution of all 12 *B.dorsalis* populations pooled together as well as of the Central China group only were distinctly unimodal (Fig. 3, 4), suggesting the further testing of a sudden expansion model.

The mismatch distribution of the 12 populations pooled together was compatible with the sudden expansion model based on concatenated sequences (P_SSD_ = 0.556) as well as each individual gene (see Tab. 3). Parameters of the expansion model were θ_0_ = 0.007, θ_1_ = 89.575 and τ = 24.453. The mismatch distribution of the Central China group was similarly compatible with the sudden expansion model based on concatenated sequences (P_SSD_ = 0.455) and individual genes (see Tab. 3). Parameters of the expansion model for the Central China group were θ_0_ = 0.019, θ_1_ = 99.248 and τ = 22.164. The sudden expansion model was in turn rejected for population groups South and South-East China (P<0.05). Individual genes (see Tab. 3) are in accord with concatenated sequences, all being compatible with the sudden expansion model for all 12 populations pooled and the Central China group (P>0.05) and most rejecting the expansion model in the other two population groups.

The ratio between estimated effective population size after expansion (θ_1_) and before expansion (θ_0_), an estimate of the extent of population growth, is 12796× for the entire dataset and 5223× for the Central China group based on concatenated sequences. Individual genes, although with differing numerical estimates, similarly suggest a large population growth, from 33× to 7144× for the entire dataset and from 85× to actually infinite for the Central China group.

## Discussion

### High level of genetic diversity

Invasive species are generally associated to a loss in genetic diversity that can take place, during an invasion process, due to a) increased genetic drift associated to the temporarily reduced population size in founding colonies [45]–[47] and b) increased selection pressure encountered during the colonization of new habitats [48].

Chinese populations of the oriental fruit fly, nevertheless, seem to retain fairly high levels of genetic diversity, as exemplified by the high observed values of haplotype diversity. Noteworthy, this is observed even in populations such as Wuhan, Xiushan, Wulong, Wanzhou and Jiangjin that, based on collection records, established not longer than a decade ago and that display values of genetic variability actually higher than some of the oldest populations in the study set, Fuzhou and Guangzhou.

Nevertheless, some characteristics of the oriental fruit fly and the ecology of the area have to be considered that could help explain why no loss in genetic variability is observed concurrent to the colonization process. Due to the high reproductive potential of this species and high dispersal capabilities, together with the relative absence of major geographical or ecological barriers to dispersal in the area, it is possible to envision how the oriental fruit fly might have gradually expanded without any significant or prolonged bottleneck. Furthermore, the relative abundance of suitable host fruits such as mango, carambola and guava, the large orange plantations in South China and Yangtze valley, and the relative uniformity and stability of a suitable tropical and subtropical monsoon climate in this area might have exerted little novel selective pressures during the range expansion in Central China.

Furthermore, multiple introductions, or colonization of a given area from multiple sources, can counteract the drop in genetic variability associated with colonization or rescue a species from an actual loss in genetic diversity [48]. Such cases of increased genetic variability due to a secondary mixing between previously diversified populations have been repeatedly described in the literature [49]–[53] and multiple introductions have been previously suggested to explain the relatively high genetic variability of the oriental fruit fly in the Yunnan province of China [13].

The oriental fruit fly may have, therefore, expanded quickly but gradually in mainland China, without the significant bottlenecks generally associated with a range expansion that takes place through invasive propagules of few individuals. This view is further in line with the high levels of gene flow and limited population differentiation observed (see below) and with the situation described by Aketarawong et al. [18] in South-East Asia and Shi [13] in the province of Yunnan.

### Weak genetic structure

Although some structure could be identified by the SAMOVA analysis, leading to the partitioning of the 12 populations studied in the three groups Central, South-East and Southern China, the overall level of differentiation was low. According to the analysis of molecular variance, more than 90% of variability was observed inside populations, with no or marginal differentiation between populations and some differences arising only between population groups. This is rather unexpected given the distances involved, with the area occupied by the Central group being roughly equivalent to Central Europe in size, but in line with other examples of highly vagile fruit fly species that have expanded in large areas in relatively recent times [28], [54].

The median joining networks for the three genes, accordingly, did not describe any underlying structure, as networks are distinctively star-like and haplotypes sampled in the three regions, not to mention populations, appear randomly distributed. The Mantel test identified a significant, though not strong, correlation between genetic and geographic distance, suggesting a certain degree of isolation by distance with no major discontinuity.

Taken together, these observations suggest a relative genetic uniformity of the oriental fruit fly in China. This is in line with the notion that the species has high dispersal capacities, with eggs and larvae that can disperse efficiently inside host fruits both under natural conditions (i.e. along rivers and ocean currents [55]) and artificially through trades of infested fruits, and adults that can fly as far as 46 km under experimental conditions [56].

It is therefore possible to hypothesize that the natural barriers present in the study area (Daba Mountains, Hengduan Mountains, Wuyi Mountains, Yangtze River, Zhujiang River and Qiongzhou Strait) are not sufficient to interrupt or determine a significant limitation to gene flow. A similar situation has been described in the region of Yunnan, where weak genetic structure was observed among 14 oriental fruit fly populations despite the presence of three big rivers and mountain ranges running North-West to South-East in the area [57]. Opposite results, i.e. the insurgence of a clear genetic structure, were in turn obtained for more sedentary species, such as the rice stem borer *Chilo suppreddalis* [58], where rivers and mountain ranges act as effective barriers to gene flow.

### Demographic history

The significant negative Tajiams'*D* and Fu's *Fs* values showed that the oriental fruit fly populations in mainland China do not fit a simple model of selective neutrality [59], [60] due to an excess in low frequency alleles. This, together with collection data that indicate a substantial increase of oriental fruit fly in terms of both geographic range and population numbers, suggested the possibility of a recent population expansion. In turn, analysis of Tajiams'*D* and Fu's *Fs* estimators separately in the three groups indicated that the Central China group may have specifically undergone a regime of population expansion. The unimodal mismatch distribution [61] for all genes in the entire dataset and specifically in the Central China group, as well as non significant SSD values against the null hypothesis of a sudden population expansion further support this hypothesis [62]. The notion that the species underwent a significant population expansion is further in line with the observation that Median Joining networks for the three genes have a distinctly star like structure, typical of expansion demographic processes.

These results are concordant with trapping data in the area. Oldest presence of the species is reported from the area facing the island of Taiwan, where the species was first detected, and the island of Hainan. These two areas correspond to population groups South-East and South China, respectively, that based on our data show no sign of population expansion. In turn, the marked range expansion observed in the last few decades mainly interested mainland China up to the 32N parallel, a very large region corresponding to population group Central China, for which distinctive signs of population expansion could be described.

Based on historical records and the genetic data presented here, it is therefore possible to reconstruct a process of fast and recent range expansion from the coastline area facing the South China sea, where the species has been likely established for a longer period of time, to a very large mainland region that has been colonized in the last few decades. Based on the high genetic diversity in the area and limited genetic structure (see above), we hypothesize that this process of colonization may be interpreted as a gradual, though fast, range expansion associated with high population numbers and population growth. The opposite scenario of a stepping stone model of repeated colonization events through numerically limited propagules may on the other the hand be excluded, as no trace of genetic bottlenecks and related drop in genetic variability is observed.

The notion that local natural barriers seem to be rather ineffective in counteracting oriental fruit fly dispersal, as well as the potential distribution analysis, suggests that the process described here of population expansion in mainland China may interest other regions north of the 32N parallel in the next decades.

### Region of origin

Different hypotheses have been proposed about the geographic origin of the oriental fruit fly. While the current distributional range of the species is rather ample, from India to Hawaii encompassing all South-East Asia, historical records clearly show that marginal populations represent recent introductions, with the species being most likely East-Asian in origin. One initial hypothesis on the geographic origin of the oriental fruit fly, based on the earliest records of its presence in the island, is Taiwan [6]. Subsequent studies, based on the high levels of local genetic variability, hypothesized Yunnan as a possible source area for the species [13], or at least an area of old colonization [57]. The most comprehensive study to date on the phylogeography of this species [18], in turn, suggested China as the source area for South-East Asian populations, and possibly for the species as a whole, based on the observation that China is equally divergent from Pacific and South-East Asian populations. Furthermore, a well supported pattern of a West-ward migration from China to colonize South-East Asia was described. Noteworthy, Aketarawong and colleagues, based on asymmetrical gene flow values, could exclude Taiwan as the source of the invasion.

Based on our extended sampling in mainland China, that greatly expands the current knowledge on local oriental fruit fly diversity beyond the region of Yunnan [13], [57] and one single population in Guangdong [18], we could further explore the patterns of diversity and range expansion in China to support an origin of the species in the coastal region facing the South China Sea, corresponding in genetic terms to the population group here described as South-East China. Main support for this hypothesis is found in the asymmetric gene flow observed from location Guangzhou (Guangdong) to 6 out of 11 alternative locations scattered in mainland China. Furthermore, this hypothesis is in line with the historical records showing that population groups South and South-East China occupy regions where the species has been present since the last century, while population group Central China, that we associate with the expansion process, occupies the mainland regions where the oriental fruit fly has been reported in the last two decades only.

Further support for the costal region of Southern China being the source area for the species as a whole comes from a joint interpretation of our results and those presented by Aketarawong et al. [18]. The two studies, taken together, cover the majority of the oriental fruit fly geographic range, and all regions that are plausible candidates for the early differentiation of the species: China and South-East Asia from Bangladesh to Hawaii. In turn, both studies describe a similar pattern of expansion from a single and the same region (Guangdong) throughout South-East Asia [18] and mainland China (this study), with a distinct signal of asymmetrical gene flow out of this region.

## Supporting Information

Figure S1 **Scatter plots of genetic distance vs. geographic distance (in ln scale) for pairwise population comparisons.** A: concatenated sequences, B: *nad1*, C: *cytb*, D: *nad5*.
(TIF)

Table S1 **Genetic diversity indices.** V, number of variable sites; n, number of unique haplotypes; H, haplotype diversity; π, nucleotide diversity; k, average number of nucleotide differences.
(DOC)

Table S2 **Estimates of population size and effective immigration rate between populations pairs.** Θ: mutation scaled effective population size; M: mutation scaled effective immigration rate. In parentheses the 95% HPD intervals. Instances of asymmetrical gene flow are indicated in bold. The source population is indicated in columns, the target population in row.
(DOC)
