# Supplementary figures and images for: The Oriental Fruit Fly, Bactrocera dorsalis, in China: Origin and Gradual Inland Range Expansion Associated with Population Growth
Source: PLoS One. 2011 Oct 3;6(10):e25238. doi: 10.1371/journal.pone.0025238 (PMC3184951; doi:10.1371/journal.pone.0025238)

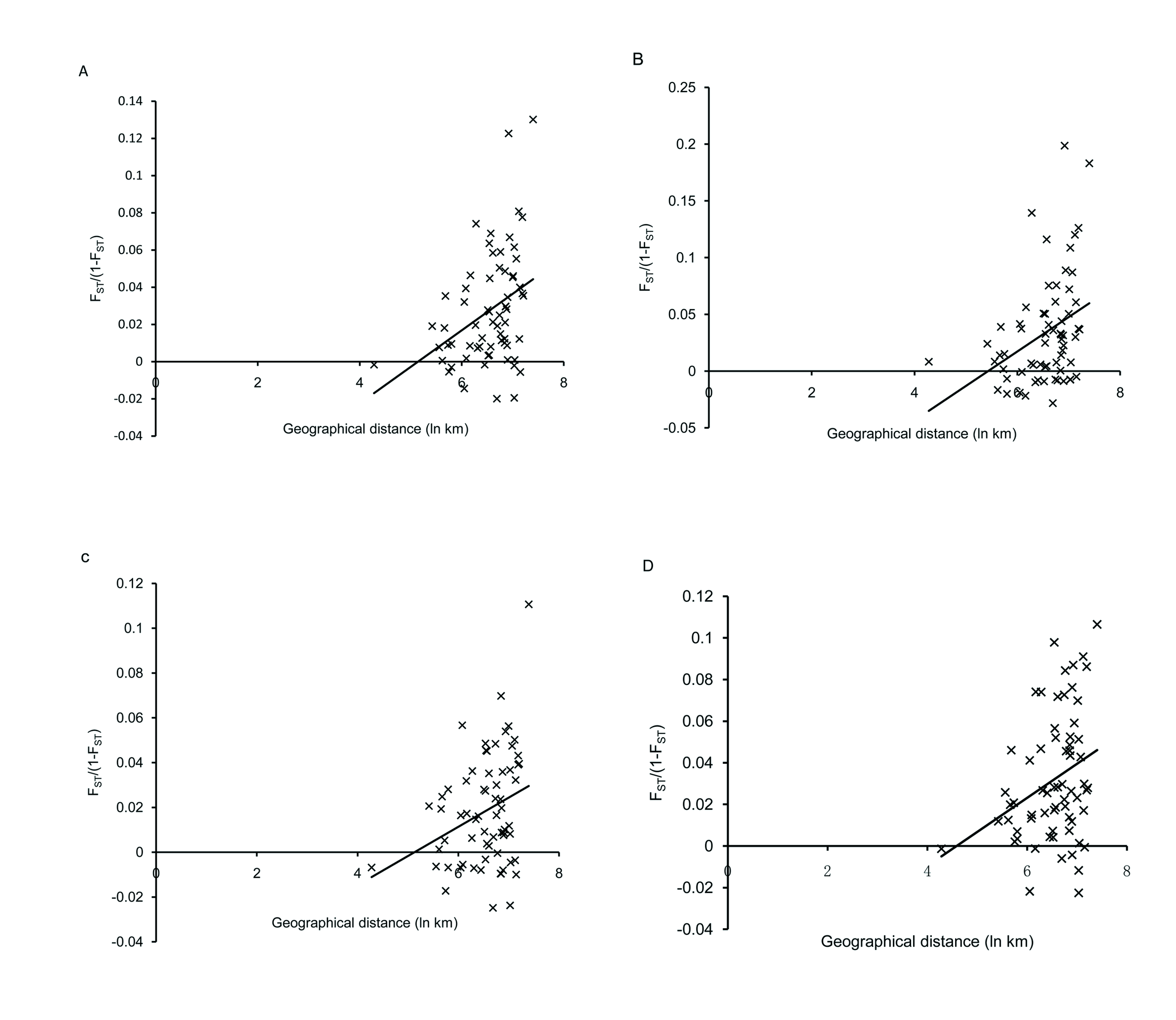

Supplement: Figure S1 — Scatter plots of genetic distance vs. geographic distance (in ln scale) for pairwise population comparisons. A: concatenated sequences, B: nad1, C: cytb, D: nad5. (TIF) [file pone.0025238.s001.tif]
