# Supplementary material for: The Oriental Fruit Fly, Bactrocera dorsalis, in China: Origin and Gradual Inland Range Expansion Associated with Population Growth
Source: PLoS One. 2011 Oct 3;6(10):e25238. doi: 10.1371/journal.pone.0025238 (PMC3184951; doi:10.1371/journal.pone.0025238)
Supplement: Table S1 — Genetic diversity indices. V, number of variable sites; n, number of unique haplotypes; H, haplotype diversity; π, nucleotide diversity; k, average number of nucleotide differences. (DOC) [file pone.0025238.s002.doc]

| Gene | Population | V | n | H | π | k |
| --- | --- | --- | --- | --- | --- | --- |
| Conc. | FZ | 69 | 14 | 0.9706 | 0.0105 | 20.6176 |
| sequences | WC | 97 | 11 | 0.9333 | 0.0123 | 24.3417 |
|  | NN | 118 | 20 | 1.0000 | 0.0115 | 22.7684 |
|  | HX | 82 | 9 | 1.0000 | 0.0124 | 24.3611 |
|  | WH | 110 | 18 | 0.9842 | 0.0106 | 20.8684 |
|  | GZ | 81 | 12 | 0.9006 | 0.0101 | 19.9415 |
|  | NC | 81 | 13 | 0.9316 | 0.0093 | 18.5158 |
|  | JS | 112 | 20 | 1.0000 | 0.0093 | 18.3526 |
|  | JJ | 164 | 19 | 0.9947 | 0.0124 | 24.5263 |
|  | WZ | 106 | 16 | 0.9684 | 0.0115 | 22.8316 |
|  | WL | 123 | 20 | 1.0000 | 0.0100 | 19.8158 |
|  | XS | 119 | 17 | 0.9842 | 0.0125 | 24.8579 |
| *nad1* | FZ | 19 | 6 | 0.9191 | 0.0092 | 5.2500 |
|  | WC | 32 | 11 | 0.9333 | 0.0147 | 8.4083 |
|  | NN | 38 | 19 | 0.9947 | 0.0116 | 6.6631 |
|  | HX | 19 | 9 | 1.0000 | 0.0102 | 5.8611 |
|  | WH | 34 | 16 | 0.9737 | 0.0111 | 6.3684 |
|  | GZ | 18 | 11 | 0.8889 | 0.0085 | 4.8538 |
|  | NC | 22 | 13 | 0.9316 | 0.0091 | 5.2368 |
|  | JS | 33 | 17 | 0.9790 | 0.0090 | 5.1632 |
|  | JJ | 26 | 17 | 0.9842 | 0.0080 | 4.6000 |
|  | WZ | 30 | 16 | 0.9684 | 0.0115 | 6.6211 |
|  | WL | 26 | 18 | 0.9895 | 0.0077 | 4.4316 |
|  | XS | 37 | 16 | 0.9737 | 0.0135 | 7.7737 |
| *cytb* | FZ | 32 | 12 | 0.9338 | 0.0126 | 9.4411 |
|  | WC | 35 | 10 | 0.9167 | 0.0118 | 8.8250 |
|  | NN | 49 | 18 | 0.9895 | 0.0132 | 9.9211 |
|  | HX | 35 | 8 | 0.9722 | 0.0144 | 10.8333 |
|  | WH | 43 | 17 | 0.9790 | 0.0112 | 8.4000 |
|  | GZ | 36 | 11 | 0.8947 | 0.0118 | 8.7251 |
|  | NC | 31 | 13 | 0.9316 | 0.0106 | 7.9158 |
|  | JS | 43 | 19 | 0.9947 | 0.0105 | 7.8842 |
|  | JJ | 102 | 19 | 0.9947 | 0.0194 | 14.5684 |
|  | WZ | 42 | 16 | 0.9684 | 0.0119 | 8.9421 |
|  | WL | 53 | 20 | 1.0000 | 0.0123 | 9.2211 |
|  | XS | 47 | 17 | 0.9842 | 0.0139 | 10.4211 |
| *nad5* | FZ | 18 | 10 | 0.9191 | 0.0090 | 5.9265 |
|  | WC | 30 | 11 | 0.9333 | 0.0108 | 7.1083 |
|  | NN | 31 | 19 | 0.9947 | 0.0094 | 6.1842 |
|  | HX | 28 | 9 | 1.0000 | 0.0117 | 7.6667 |
|  | WH | 33 | 17 | 0.9790 | 0.0093 | 6.1000 |
|  | GZ | 27 | 11 | 0.8947 | 0.0097 | 6.3626 |
|  | NC | 28 | 13 | 0.9316 | 0.0082 | 5.3632 |
|  | JS | 36 | 19 | 0.9947 | 0.0081 | 5.3053 |
|  | JJ | 36 | 19 | 0.9947 | 0.0081 | 5.3579 |
|  | WZ | 34 | 15 | 0.9632 | 0.0111 | 7.2684 |
|  | WL | 44 | 20 | 1.0000 | 0.0094 | 6.1632 |
|  | XS | 35 | 17 | 0.9842 | 0.0101 | 6.6632 |
