# Supplementary material for: The Oriental Fruit Fly, Bactrocera dorsalis, in China: Origin and Gradual Inland Range Expansion Associated with Population Growth
Source: PLoS One. 2011 Oct 3;6(10):e25238. doi: 10.1371/journal.pone.0025238 (PMC3184951; doi:10.1371/journal.pone.0025238)
Supplement: Table S2 — Estimates of population size and effective immigration rate between populations pairs. Θ: mutation scaled effective population size; M: mutation scaled effective immigration rate. In parentheses the 95% HPD intervals. Instances of asymmetrical gene flow are indicated in bold. The source population is indicated in columns, the target population in row. (DOC) [file pone.0025238.s003.doc]

| Pop. | Θ | Fuzhou | Wenchang | Nanning | Huaxi | Wuhan | Guangzhou | Nanchang | Jianshui | Jiangjin | Wanzhou | Wulong | Xiushan |
| --- | --- | --- | --- | --- | --- | --- | --- | --- | --- | --- | --- | --- | --- |
| FZ | 0.03153 (0.00380- 0.06771) | - | 489.8 (20.7- 509.3) | 376.6 (52.7- 692.0) | 150.5 (0.0- 394.7) | 204.4 (25.3- 448.0) | **853.0 (576.0- 1000.0)** | 407.4 (91.3- 722.0) | 333.2 (80.7- 630.0) | 229.7 (5.3- 689.3) | 313.8 (70.7- 270.3) | 336.3  (54.7- 283.7 | 314.5 (45.3- 632.7) |
| WC | 0.00916 (0.00320- 0.01528) | 670.2 (326.7- 1000.0) | - | 552.6 (166.0- 972.3) | 487.4 (126.0- 870.0) | 299.4 (0.0- 628.7) | **707.0 (347.3- 1000.0)** | 335.6 (51.3- 670.7) | 472.9 (75.3- 862.7) | 515.4 (184.7- 903.3) | 380.2 (93.3- 670.0) | 281.6 (32.7- 558.7) | 427.6 (116.7- 868.7) |
| NN | 0.08087 (0.05290- 0.10000) | 598.7 (273.3- 994.7) | 371.0 (25.3- 721.3) | - | 282.2 (0.0- 685.3) | 442.0 (127.3- 757.3) | 348.3 (56.7- 676.0) | 503.4 (218.0- 866.7) | 625.7 (329.3- 1000.0) | 581.6 (417.3- 967.3) | 399.4 (24.7- 700.7) | 457.6 (43.3- 828.0) | 369.9 (55.3- 749.3) |
| HX | 0.07941 (0.05063- 0.10000) | 442.8 (106.0- 844.0) | 418.0 (70.7- 780.0) | 536.0 (230.0- 892.0) | - | 537.8 (218.0- 900.0) | 470.7 (112.7- 906.7) | 537.5 (203.3- 895.3) | 541.2 (146.7- 894.7) | 492.2 (124.7- 914.7) | 538.0 (192.0- 892.0) | 532.2 (202.0- 944.0) | 580.2 (370.0- 996.7) |
| WH | 0.07351 (0.03789- 0.10000) | 352.5 (44.0- 695.3) | 402.3 (36.7- 797.3) | 462.3 (181.3- 779.3) | 500.8 (60.0- 911.3) | - | **584.9 (256.7- 1000.0)** | 446.5 (50.7- 854.7) | 416.1 (88.7- 714.7) | 430.1 (119.3- 806.0) | 463.2 (149.3- 801.3) | 313.4 (64.7- 281.0) | 395.5 (88.0- 814.7) |
| GZ | 0.00368 (0.00127- 0.00370) | **211.5 (30.0- 457.3)** | **253.3 (68.0- 494.7)** | 158.6 (24.0- 336.0) | 125.6 (0.0- 305.3) | **96.6 (0.0- 242.7)** | - | 143.7 (19.3- 288.7) | 176.4 (22.7- 343.3) | **124.6 (16.0- 246.7)** | **121.7 (0.0- 285.3)** | 189.7 (55.3- 357.3) | **87.6 (0.0- 192.7)** |
| NC | 0.03119 (0.00400- 0.06765) | 559.8 (144.0- 912.7) | 357.3 (85.3- 615.3) | 410.5 (49.3- 778.0) | 567.7 (237.3- 958.0) | 278.9 (56.0- 622.0) | 796.1 (494.7- 1000.0) | - | 382.8 (62.7- 822.0) | 298.4 (48.0- 639.3) | 465.3 (70.7- 854.7) | 377.3 (82.7- 765.3) | 189.6 (20.0- 410.0) |
| JS | 0.07973 (0.05030- 0.10000) | 492.6 (168.0-  880.0) | 293.6 (0.0- 615.3) | 443.5 (151.3- 792.0) | 648.1 (266.7- 996.7) | 483.3 (81.3- 886.0) | 507.5 (184.0- 914.0) | 467.2 (116.7- 938.0) | - | 348.4 (33.3- 766.0) | 459.2 (169.3- 773.3) | 518.0 (180.0- 882.0) | 484.4 (147.3- 907.3) |
| JJ | 0.07948 (0.05197- 0.10000) | 500.3 (86.0- 862.0) | 385.5 (71.3- 767.3) | 569.4 (257.3- 930.0) | 284.3 (26.7- 608.7) | 404.2 (64.7- 807.3) | **712.0 (370.7- 1000.0)** | 397.5 (55.3- 878.0) | 374.3 (98.7- 736.0) |  | 533.1 (183.3- 914.0) | 401.8 (98.7- 782.0) | 204.7 (14.0- 184.3) |
| WZ | 0.04481 (0.01501- 0.08466) | 507.0 (176.0- 966.7) | 333.6 (142.0- 555.3) | 333.6 (38.0- 684.7) | 493.0 (118.0- 469.0) | 655.2 (262.7- 691.0) | **643.0 (314.0- 643.7)** | 504.2 (141.3- 870.7) | 333.8 (100.0- 582.7) | 467.1 (72.7- 916.0) | - | 405.0 (154.0- 678.0) | 368.8 (104.7- 714.7) |
| WL | 0.08317 (0.05844- 0.10000 | 325.1 (36.0- 702.7) | 250.3 (24.7- 580.70) | 350.8 (88.0- 279.7) | 352.4 (78.0- 728.0) | 349.8 (92.7- 655.3) | 681.3 (354.0- 944.7) | 398.6 (87.3- 755.3) | 529.5 (223.3- 907.3) | 370.2 (48.7- 773.3) | 332.2 (69.3- 604.0) | - | 374.2 (23.3- 620.7) |
| XS | 0.06170 (0.03122- 0.09967) | 442.9 (86.7- 784.0) | 476.7 (131.3- 847.3) | 288.8 (36.0- 545.3) | 273.9 (0.0- 683.3) | 459.5 (122.0- 836.7) | **540.8 (207.3- 536.3)** | 324.6 (76.0- 684.0) | 443.2 (128.7- 782.0) | 462.0 (130.0- 779.3) | 460.2 (104.0- 886.0) | 445.9 (24.0- 877.3) | - |
